# CIRSE Standards of Practice on the Endovascular Management of Descending Thoracic Aortic Disease

**DOI:** 10.1007/s00270-026-04477-5

**Published:** 2026-08-03

**Authors:** Yuri Gupta, Samuel Vaughan, Fabrizio Fanelli, Catharina van Rijswijk, Hervé Rousseau, Jost Philipp Schäfer

**Affiliations:** 1 University Hospitals Sussex, Brighton, UK; 2 Vascular and Interventional Radiology Department, Careggi’ University Hospital, Florence, Italy; 3 Leids Universitair Medisch Centrum, Leiden, Netherlands; 4 CHU Rangueil, Cedex 9 Toulouse, France; 5 University Hospital Schleswig Holstein Campus Kiel, Kiel, Germany

**Keywords:** TEVAR, Aorta thoracic, Aortic aneurysm thoracic, Aortic dissection, Standards of Practice

## Abstract

Endovascular management of descending thoracic aortic disease (aneurysm, acute aortic syndrome and traumatic injury) is an effective, minimally invasive treatment that over the last 20 years has superseded surgical repair and become the standard of care. This CIRSE Standards of Practice document recommends best evidence-based practices for the endovascular management of descending thoracic aortic disease. This includes diagnostic imaging, surveillance, indications for intervention, endovascular treatments, their outcomes, and follow-up. The writing group, established by the CIRSE Standards of Practice Committee, reviewed the literature, and the final recommendations were formulated through consensus. Endovascular treatment has an established role in the successful management of thoracic aortic disease, and this Standards of Practice document provides up‐to-date recommendations for its safe performance.

## Introduction

The CIRSE Standards of Practice Committee established a writing group, which was tasked with producing up‐to-date recommendations for the endovascular management of descending thoracic aortic disease. This CIRSE Standards of Practice document recommends best practices for, and a reasonable approach to, the endovascular management of descending thoracic aortic disease. CIRSE Standards of Practice documents are neither clinical practice guidelines nor systematic reviews of the literature, nor are they intended to impose a standard of clinical patient care. The current document updates the 2009 CIRSE SOP for the Endovascular Treatment of Thoracic Aortic Aneurysms (TAAs) and Type B Dissections [1]. Institutions should regularly review their internal procedures for development and improvement, taking into account contemporary international guidance, local resources, and regular internal morbidity and mortality reviews. A summary of key recommendations for the endovascular management of descending thoracic aortic disease can be found in Table 1.

**Table 1:** Key recommendations for the endovascular management of thoracic aortic disease

Indications for endovascular treatment of DTAD	In patients suitable for both, TEVAR is preferred over surgery (reduced morbidity, mortality, and length of hospital stay)
	**Symptomatic patients:**
	• Ruptured TAA
	• IMH/PAU with refractory pain, haemodynamic instability, imaging progression
	• TBAD with complications (refractory pain, uncontrollable hypertension, visceral/limb ischaemia, paraplegia/paraparesis, aortic rapid expansion)
	**Asymptomatic patients:**
	• Aneurysm with diameter ≥ 60 mm
	• PAU with imaging risk factors for growth (depth > 10 mm, diameter > 20 mm)
	• TBAD with late complications on surveillance
	**BTAI:**
	• Grade 2–3 injury (pseudoaneurysm, rupture)
Patient preparation	• Pre-procedure Anaesthetic review (preferably for general anaesthesia)
	• Imaging: CTA of entire aorta, including head, neck (for vertebral anatomy), and femoral arteries, with ≤ 1 mm slices and multiplane reconstructions
	• Landing zones: Minimum 15–25 mm proximal/distal sealing zones. Consider covering ± revascularisation of branch vessels
	**Device sizing:** (relative to adjacent normal aorta)
	• Diameter: DTAA: 15–20% oversize, TBAD: 0–10% oversize
	• Length: Measure aorta outer curvature for length of coverage
Treatment	Consider pre-emptive LSA revascularisation if LSA coverage is required
	**Stent graft deployment:**
	• Stiff wire to ascending aorta
	• Oblique angiography (usually left anterior) of proximal landing zone
	• Deploy stent graft with cardiac output reduction (if systolic pressure > 100 mmHg)
	• Multiple stent grafts: deploy proximal to distal in TBAD (to cover entry tear first), deploy smaller diameter device before larger
	• Dilate the stent graft using a compliant balloon (only for aneurysms, not dissections) ‐ consider artificial tachyarrhythmia to avoid stent graft migration
	Final angiography to confirm positioning, branch perfusion, true/false lumen perfusion in TBAD, and check for endoleak
Follow-up	CTA at 1 and 12 months (plus a 3- to 6-month scan for dissection, or if 1-month scan is abnormal) and then annual scans (if stable after 3 years, can extend to 2–3 yearly)

*DTAD* Descending thoracic aortic diseases; *TAA* Thoracic aortic aneurysm*; IMH* Intramural haematoma; *TBAD* Type B aortic dissection*; PAU* Penetrating aortic ulcer; *BTAI* Blunt traumatic aortic injury; *DTAA* Descending thoracic aortic aneurysm; *LSA* Left subclavian artery

## Methods

The writing group, which was established by the CIRSE Standards of Practice Committee, consisted of five clinicians with internationally recognised expertise in endovascular management of descending thoracic aortic disease and a research assistant (SV). The writing group reviewed existing literature on the subject, performing a pragmatic evidence search using PubMed to select relevant publications in the English language and involving human subjects, preferably published from 2009, following publication of the initial SOP on this topic, to 2026. Relevant older sources were also included where the data have not been updated and clear gaps were evident. The final recommendations were formulated through consensus.

## Background

Descending thoracic aortic diseases (DTADs) treated by endovascular repair include descending thoracic aortic aneurysm (DTAA), acute aortic syndrome (AAS), and blunt traumatic aortic injury (BTAI).

### Descending Thoracic Aortic Aneurysm

The descending thoracic aorta (DTA) is the third most common location for aortic aneurysms (after the abdomen and ascending aorta), with an incidence estimated at 6–10.4 per 100,000 person-years [2]. The apparent incidence is increasing in our ageing general population with increased awareness and increased use of, and improvements in, diagnostics. The 5-year survival rate for patients with an untreated DTAA > 60 mm has been reported as 54%, with an annual rupture risk of 3.7% and mortality risk of 11.8% [3]. Significant risk factors for rupture include aortic diameter, pain, older age, and chronic obstructive pulmonary disease. DTAAs can be classified according to shape and extent. The aneurysm shape can be fusiform or saccular (the latter typically starting as a post-traumatic pseudoaneurysm or penetrating aortic ulcer (PAU)). Although less common than in abdominal aortic aneurysms, DTAA can also become infected (Swedish vascular registry data showed that 3.6% of patients undergoing DTAA repairs were for mycotic disease [4]), which carries an increased risk of rupture.

### Acute Aortic Syndrome

AAS comprises a spectrum of three interrelated diseases: aortic dissection, PAU, and intramural haematoma (IMH). Key risk factors include age, male gender, hypertension, smoking, aortic aneurysm, and genetic disorders including Marfan syndrome. Dissection and PAU can result from ulceration of a pre-existing atherosclerotic plaque, while IMH can develop in isolation, and both IMH and PAU can lead to dissection. AAS may be complicated by end-organ ischaemia, aneurysmal transformation, or rupture. Early diagnosis is critical to allow timely and appropriate management.

The prevalence of PAU amongst symptomatic AAS patients is 2.3–7.6%, 90% of which are within the DTA [5]. IMH accounts for 5–20% of patients with AAS, involving the DTA in 60% of cases [6].

Aortic dissection is classified according to location (the Stanford classification is widely used [7]): type A is defined as any dissection involving the ascending aorta, type B when only the descending aorta is involved, and non-A-non-B in cases of arch involvement by the most proximal tear or by retrograde extension [8]. These guidelines also define three phases of aortic dissection: acute 1–14 days, subacute 15–90 days, and chronic after 90 days. Type B aortic dissection (TBAD) most commonly affects male patients and has an annual incidence of up to 15 per 100,000 population [9, 10]. 60–75% of TBAD patients present with uncomplicated disease (uTBAD) [11, 12]; however, these patients still have an in-hospital mortality of 3–10% [13–15], due to late complications. Nearly 40% of patients with uTBAD require late intervention, mostly for aneurysmal degeneration, with long-term survival of 50–60% [13–15]. Patients with complicated TBAD (cTBAD), defined as the presence of refractory pain or uncontrollable hypertension (despite best medical therapy), clinical signs of visceral/limb ischaemia, paraplegia/paraparesis, and aortic rapid expansion or rupture, have a mortality of 20% by day 2, and 25% by day 30 [16].

### Blunt Traumatic Aortic Injury

Trauma is the leading cause of death for people in the first four decades of life, and of these BTAI is second most common [17], causing sudden death in 80–90% of patients [18]. Injury can occur anywhere in the aorta, but occurs most commonly at the isthmus (55–90%) [17, 19]. Several classification systems have been described to grade aortic injury severity. The most recent European guidance describes three grades: 1—partial wall injury (intimal flap, IMH); 2—contained complete wall injury (pseudoaneurysm); and 3—complete wall transection (rupture). A small proportion of BTAI may go undetected initially (2–8%), with 50% of these developing late symptoms and 20% mortality due to rupture within 15 years of injury [20].

### Indications, Contraindications, and Patient Selection

#### Descending Thoracic Aortic Aneurysm

Either open surgery or thoracic endovascular aortic repair (TEVAR) is indicated when there is clear evidence of aortic rupture, or when patients present with symptoms and/or an aortic diameter ≥ 60 mm [21], including those with evidence of mycotic DTAA. In patients suitable for both TEVAR or surgery (based on comorbidities and performance status), TEVAR is preferred given its reduced morbidity, mortality, and length of hospital stay [22]. Open surgery is recommended in the rarer cases that involve connective tissue diseases [18]. Discussion of endovascular repair of aneurysms involving both the descending thoracic and abdominal aorta lies beyond the scope of this article.

#### Acute Aortic Syndrome

TEVAR is indicated in symptomatic IMH and PAU with refractory pain or haemodynamic instability, or disease progression on imaging despite best medical therapy. Asymptomatic IMH is usually managed conservatively with close observation and blood pressure control. Evidence regarding asymptomatic PAU is limited, but guidelines suggest treating with TEVAR when there are risk factors for growth or rupture on imaging (PAU maximum depth > 10 mm, or diameter > 20 mm) [22].

Type A dissection has a high risk of complications and is usually managed with open surgery. Discussion of the endovascular management of infrequently encountered non-A-non-B cases (which are increasingly treated with endovascular or hybrid approaches in specialised centres) lies beyond the scope of this article. Patients presenting with uTBAD are initially managed with best medical therapy (including pain relief and strict blood pressure control), which aims to prevent progression of the dissection and rupture.

TEVAR is the current standard of care for cTBAD due to the dramatically improved outcomes and reduced mortality compared with open surgery [23]. The objective is to cover the entry tear and so reduce blood pressure within the false lumen, which can prevent antegrade or retrograde propagation of the dissection and lead to false lumen thrombosis and later aortic wall stabilisation and remodelling. The clinical goal is the prevention and treatment of aortic rupture and malperfusion syndromes. Management strategies are therefore complication and anatomy specific, guided by the patient’s signs and symptoms and the presence or absence of complications.

#### Trauma

Grade 1 injuries (intimal flap or IMH) carry a low risk of rupture and may be managed expectantly, with priority given to managing other life-threatening injuries. Patients with grade 2–3 injuries (pseudoaneurysm and rupture) require emergent repair [18] (which may be immediate or delayed depending on concomitant injuries and haemodynamic status).

Similar to DTAA and TBAD, TEVAR for BTAI carries a significantly lower mortality risk than open surgery (9.7% vs. 27.7%), with lower paralysis risk (trend 0.4% vs. 2.9%) but a higher stroke risk (2.3% vs. 0.4%) [24], and it has become the first choice of treatment for BTAI.

## Patient Preparation

Where possible, the procedure, complications, and success rate should be discussed by the treating physician in an outpatient setting prior to the procedure, and informed consent should be obtained [25, 26].

### Pre-procedural Imaging

All patients being managed for DTAD require arterial phase contrast-enhanced CTA of the entire aorta including iliac and femoral arteries, with ≤ 1 mm slice thickness, in conjunction with post-acquisition image processing and multiplane reconstruction software. The use of electrocardiographic-gated scanning is advised for aortic root and ascending aortic pathology to minimise pulsation artefacts. CTA of the head and neck should be included to determine the anatomy of the vertebral arteries and circle of Willis, especially when covering of the left subclavian artery (LSA) is intended. CTA has distinct advantages over other imaging options including availability and speed of imaging, ease of interpretation, fewer artefacts, and sizing accuracy.

Diameter measurements of the aortic arch and DTA should be performed perpendicular to the long axis of the aorta from outer edge to outer edge. Transaxial diameter measurements should not be used as they can overestimate the aortic diameter at levels of curvature and tortuosity. CTA is also the preferred imaging modality to assess vessel wall calcifications, plaque, and thrombus in landing zones, which may compromise the fixation or seal of the endograft. Length of aortic coverage is also evaluated on preoperative CTA. Excessive tortuosity in aneurysmal segments may result in erroneous length measurements because the path of the stiff wire and endograft position may not follow the centreline. Taking this into account, the outer curvature length should be used, as it has been shown to be more accurate than the centreline in planning length of aortic coverage [27].

MRA is not used routinely, given the advantages of CTA outlined above; however, MRA can provide morphologic and blood flow information without the use of iodinated contrast material or radiation exposure. MRI is therefore often a primary option for assessing congenital aortic abnormalities and is well suited for serial imaging in younger patients and those with genetic aortic syndromes and aortic inflammation [28].

### Procedure Planning

Depending on the choice of stent graft, the proximal and distal landing zones should be sufficiently long to create a minimum 15–25 mm sealing zone of healthy aorta, defined as having a uniform, non-aneurysmal diameter over a straight segment of the vessel and being relatively free of calcification or thrombus [22].

In patients with aneurysms, the proximal stent graft is typically oversized by 15–20% (relative to adjacent normal aorta) to maximise the radial force at the seal zone. Minimal oversizing is recommended in patients with aortic dissection [29, 30]. A degree of proximal oversizing in dissection patients can help ensure adequate stent graft sealing and prevention of migration; however, excessive oversizing can result in progression of the dissection, retrograde dissections, proximal or distal stent graft-induced new entry tears (SINE), and secondary aortic expansion with subsequent rupture. The presence of a dissected wall in a dilated aorta can make it difficult to assess the pre-dissection reference aortic diameter. In this situation, the aortic arch diameter just proximal to the dissected segment can be used as the reference diameter [31] and the chosen endograft is oversized relative to this by up to 10%. Special consideration must also be given to the morphology of the proximal landing zone. It may be challenging to achieve a proper seal along the inner curve of a severely angled aortic arch, which can result in a ‘bird-beak’ sign with possible type 1a endoleak, or even in-folding and graft collapse. For aortic pathologies with critically short landing zones, the recent introduction of thoracic branched stent grafts as off-the-shelf devices has increased the treatment options. Thus, the sealing zone is extended into the aortic arch to increase the proximal landing zone and to exclude the pathology while simultaneously maintaining the perfusion of the respective supra-aortic branch.

There are few recommendations on sizing of the distal landing zone, but the maximum true lumen diameter (without oversizing) is often used. There can be further challenges at this level, especially in patients with malperfusion and true lumen collapse. As the distal stent graft often lands in dissected aorta, tapered stent grafts can be employed to avoid oversizing.

**Fig. 1:**
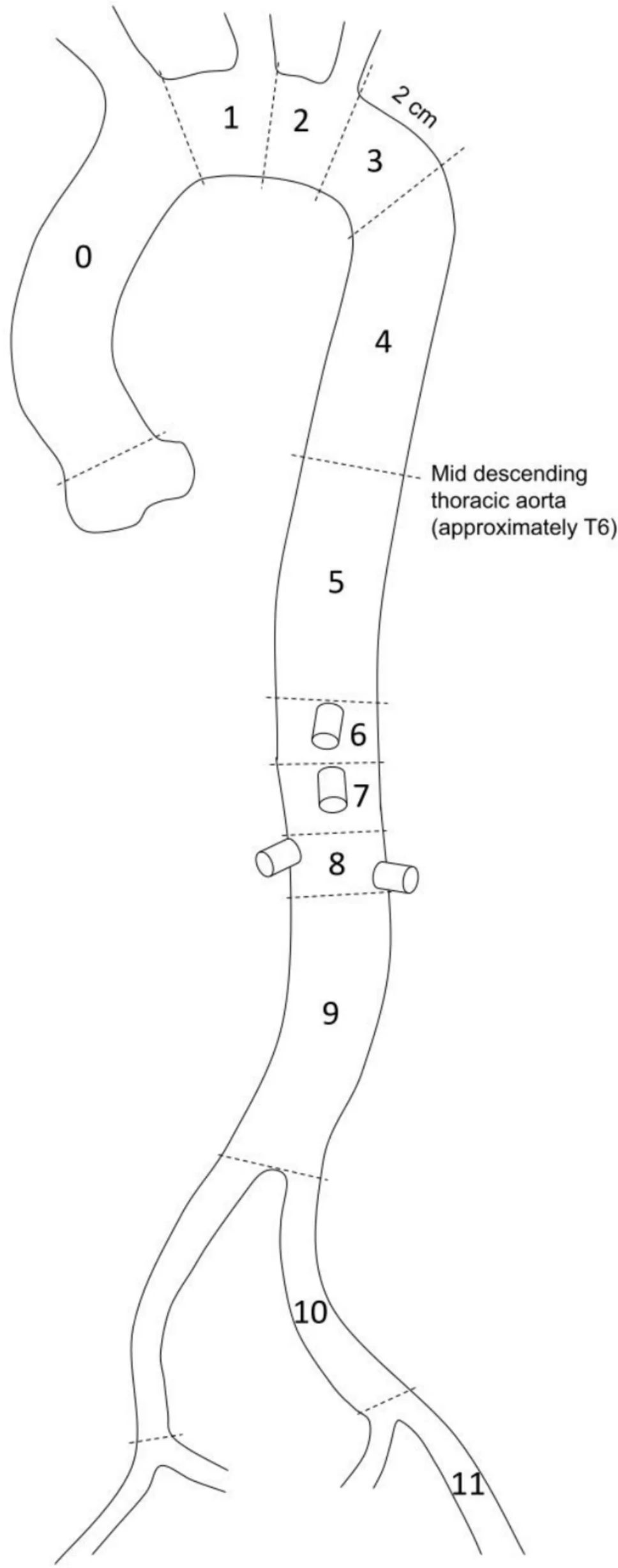
Aortic zones of attachment

Dissection, trauma, and aneurysmal disease involving the proximal DTA all frequently require proximal TEVAR positioning in zone 2 of the aorta (see Fig. 1), with covering of the LSA. This is acceptable in an emergency but can have more far-reaching implications than simply reducing blood flow to the left arm (left arm ischaemia is a rare complication of LSA coverage, due to compensatory blood flow through the circle of Willis with reversal of flow down the left vertebral artery into the arm). More important are the risks of vertebrobasilar insufficiency and spinal cord ischaemia (SCI), with resultant paraparesis or even permanent paraplegia. Pre-emptive LSA revascularisation may decrease the rates of stroke and SCI [32]. Patients with a dominant left vertebral artery, a diseased circle of Willis, left internal mammary artery bypass graft to coronary artery, or who are otherwise reliant on inflow from the LSA (e.g. for dialysis access), should also undergo LSA revascularisation.

Lastly, the access sites and iliac vessels must be assessed to ensure that endovascular repair can be safely performed. Over the last two decades, completely percutaneous TEVAR has become established with the use of percutaneous closure devices. A diverse armamentarium of such devices allows for different pre- and post-close techniques. Percutaneous access is very well tolerated by patients and allows a comfortable recovery period with early mobilisation and return to normal activities.

Stenotic and tortuous iliac vessels must also be identified and planned for in advance, and careful balloon dilation (or use of serial dilators) can be used to accommodate insertion of the large diameter devices. If the femoral or external iliac arteries are too small or severely diseased, a surgical approach to the common iliac arteries or infrarenal aorta with arteriotomy, or establishing a temporary conduit, can facilitate insertion of the devices. A brachio-femoral through-and-through wire may be needed in extreme aorta-iliac tortuosity. Alternative access sites, for example via subclavian or carotid arteries or the ascending aorta, are usually reserved for centres specialised in specific cases.

### Laboratory Workup: Anaesthetic Review, Bloods

TEVAR is ideally performed under general anaesthesia, which allows blood pressure control during stent graft deployment, and thus, pre-procedural anaesthetic review is recommended to assess the patient’s performance status, comorbidities, and suitability for anaesthesia. TEVAR can also be performed under local or regional (epidural or spinal) anaesthesia with anaesthetic support, depending on the patient’s condition and the complexity of the procedure. This approach provides the added benefit of early recognition of neurologic complications.

Laboratory workup should include a full blood count, coagulation screen, renal function assessment, and blood typing.

## Endovascular Treatment

TEVAR must be performed in sterile conditions in an angiography suite or a hybrid operating room. The use of an adequate fluoroscopy system is mandatory to provide optimal projection planes of the angiograms in different oblique views to clearly delineate the landing zones.

### Antibiotic Prophylaxis

Prophylactic antibiotics are routinely used before thoracic aortic endografts (although evidence to support the practice is limited) [33] and are usually administered shortly before starting the procedure. The Joint Commission 2011 Hospital Patient Safety Goals specify that intravenous antimicrobial prophylaxis should be administered within 1 hour before incision or within 2 hours for the administration of vancomycin and that prophylactic antimicrobial agents should be discontinued within 24 hours of the procedure unless clinical circumstances require continuation of a prophylactic regimen as therapy [34]. The most common prophylactic regimes are based on intravenous cefazolin, vancomycin, or clindamycin [33].

### Access

The contralateral femoral, brachial, or radial artery is accessed using ultrasound, a small sheath placed, and a 4–5 Fr graduated-marker pigtail catheter is advanced up to the ascending aorta or aortic arch. The aortic stent graft (18–25 Fr) is usually inserted via femoral access; however, ilio-femoral disease may necessitate an initial surgical approach (as previously described).

### Stent Graft Deployment

Before inserting the large diameter stent graft device, the patient is anticoagulated (with heparin, dosed according to body weight), and a soft guidewire and angled catheter is advanced to the ascending aorta. The soft guidewire is then exchanged for a stiff guidewire for adequate support to manoeuvre the stent graft to the target location. In cases of aortic dissection, it is mandatory to ensure passage in the true lumen, and it may be helpful to gently advance a pigtail catheter through the dissection zone within the true lumen to avoid traversal into the false lumen. The stent graft is prepared by flushing the delivery device with saline (according to the device instructions for use), to expel residual air in the system.

A pigtail catheter is advanced to the aortic arch via groin or arm access, and digital subtraction angiography should be performed in oblique projections (usually left anterior) to obtain precise information on the aortic morphology and pathology, which is then correlated with the pre-procedural CTA (or MRA). This is especially important in aortic dissections to assess the haemodynamic perfusion state via true and false lumens. The exact angulation of the C-arm can be planned from multiplane reformatting of the pre-procedure CTA/MRA, which is of utmost importance if the proximal landing zone is within the aortic arch or the distal landing zone is near the coeliac trunk. Additionally, catheters or wires can be left in the respective arteries, such as the LSA or the coeliac trunk, to serve as precise landmarks for the landing zones (particularly useful in aortic dissection). This can also be achieved by using a left trans-brachial or trans-radial approach for placing the diagnostic pigtail catheter in the aortic arch. When the constrained stent graft device is in the deployment position, further angiography should be performed to precisely delineate the anatomy and morphology of both proximal and distal landing zones.

Once the final position has been confirmed, the stent graft is carefully deployed (according to the device instructions for use), avoiding antegrade migration of the stent graft during deployment, known as the ‘windsock’ effect (described below; certain devices have a stepwise release mechanism designed to mitigate this risk). Balloon dilation of the stent graft with a compliant balloon is reserved for aneurysms, while it is contraindicated in dissection cases (due to the higher risk of aortic wall injury).

The pigtail catheter is then repositioned within the stent graft, and final angiography is performed to document the positioning of the devices, check perfusion of supra-aortic branches (especially if these have been covered) and perfusion of the true versus false lumen in aortic dissection, and exclude endoleaks in aortic aneurysm.

### Technical Considerations

#### Landing zones

In critical proximal landing zones within the aortic arch, several methods can be employed to minimise the impact of the windsock effect and facilitate precise deployment of the stent graft (detailed in the peri-procedural care section below). Bare struts at the device’s proximal end provide enhanced stability within the proximal landing zone in aortic aneurysms, while devices without a proximal bare stent are preferred in aortic dissections because the bare stent may risk further injury to the already diseased aortic wall. Severe kinking in the proximal landing zone may be addressed by using a conformable stent graft. Critical proximal landing zones may be post-ballooned under artificial tachyarrhythmia to avoid secondary stent graft migration.

#### Multiple stent grafts

When deploying more than one stent graft, there are further considerations: 1) in dissections, it is advised to deploy devices from proximal to distal, in order to cover the main proximal entry tear first; 2) with stent grafts of different diameters, the smaller distal device must be deployed first followed by the larger, with at least 5 cm or two stents overlap (to avoid type III endoleaks). Other deployment sequences, e.g. proximal or distal device first, can be undertaken dependent on the chosen devices, the user’s preference and experience, and the challenges of the landing zones.

#### Branches

Intentional covering of supra-aortic branches or the coeliac trunk may be necessary to increase the length of landing zone (as discussed in the planning section). Adjunctive prophylactic embolization of covered arteries can be performed to prevent type 2 endoleaks.

In managing branch vessel occlusion and malperfusion, endovascular techniques aim to restore branch vessel flow by addressing dynamic or static obstructions. TEVAR is the primary intervention; by sealing the entry tear, it re-expands the true lumen and depressurises the false lumen, usually resolving dynamic flap prolapse. For refractory dynamic cases, endovascular fenestration can be employed to create a flap re-entry point to equalise true and false lumen pressures. Static obstructions may require branch vessel stenting to bypass a fixed narrowing or flap extension.

Other advanced strategies include the PETTICOAT technique, which uses a distal bare-metal stent to support the true lumen across the visceral segment. The STABILISE technique builds on this, utilising balloon-induced flap disruption to achieve a single channel aorta [35]. These techniques may help facilitate rapid organ reperfusion, reducing the high mortality associated with mesenteric or renal ischaemia while promoting long-term favourable aortic remodelling, although more evidence is needed on their long-term outcomes.

## Peri-procedural Care and Follow-up

### Cerebrospinal Fluid Drainage

Paraplegia is one of the most serious complications of thoracic aorta repair, with TEVAR carrying a 2–15% risk of spinal cord injury, which adversely affects both quality of life and survival [22, 36]. Risk factors include distal landing in zones 5–10, coverage near the coeliac trunk, extensive aortic coverage (> 15–20 cm), prior aortic surgery, urgent procedures, smoking, and renal disease. Intraprocedural neuromonitoring can be used to help detect spinal cord injury risk [37, 38].

In patients with new neurological signs post-TEVAR, cerebrospinal fluid (CSF) drainage is used as a therapeutic intervention to improve spinal cord perfusion by reducing CSF pressure. Mean arterial pressure (MAP) should also be maintained above 85 mmHg for 7 days after injury [18, 22, 39] (spinal cord perfusion pressure being equal to MAP minus CSF pressure). Prophylactic CSF drainage has also been reported but, given that the procedure itself carries a risk of complications (6.5%) [40], this is not routinely recommended.

### Cardiac Output Reduction

Accurate stent graft deployment is critical to the success of the procedure. An important factor influencing precise deployment is the windsock effect. When the stent graft is opened, the aortic blood flow is completely blocked by the partially deployed stent graft, resulting in an abrupt increase in pressure on the stent graft, potentially resulting in antegrade graft displacement [41]. Decreasing the aortic impulse by decreasing the mean blood pressure is an effective way to minimise the adverse impact of the windsock effect.

Several methods can be employed to reduce the aortic impulse, including rapid right ventricular pacing (to 160–200 bpm, which reduces left ventricular filling time and hence cardiac output), pharmacological cardiac output reduction (aiming for a mean blood pressure of 60 mmHg), or inferior vena cava balloon occlusion to reduce venous inflow [41–44]. The Munich Valsalva implantation is an alternative method that avoids the use of additional medications, vascular access, or electrophysiological manipulation [43, 44]. It is based on a modified Valsalva manoeuvre in the intubated patient. An increase in intrathoracic pressure reduces venous inflow and cardiac preload, resulting in a reduction in cardiac output and a fall in systolic pressure.

### Antithrombotic therapy

Long-term single antiplatelet therapy is advised post-TEVAR to reduce the risk of future cardiovascular events [45]**.** Anticoagulation is reserved for patients with evidence of thromboembolic complications and low bleeding risk.

### Imaging Follow-up

Post-TEVAR imaging surveillance is essential to monitor procedural success, complications, and disease progression [46, 47].The follow-up protocol is based on CTA at 1 and 12 months (with additional scans at 3–6 months for TBAD or if an abnormality is seen at 1 month), followed by annual evaluation for life [18, 30, 46, 48]. After three years, in stable cases, the imaging interval can be extended to 2 years. MRA is a valid alternative and is compatible with most stent grafts; however, its use is limited by longer acquisition times and susceptibility to artefacts, particularly with stainless steel devices. MRA is therefore only recommended where CTA is contraindicated.

## Outcomes of Thoracic Aortic Stent Grafting

### Technical Success

Technical success, defined as exclusion of the relevant pathology (the aneurysmal sac, primary entry tear in dissection, or wall defect in trauma), and demonstrated by graft-directed flow without endoleak on angiography, is high (91.9–100%) [49–51]. Initial stent deployment that does not meet the criteria of a technical success will be evident on intraprocedural imaging and can in most cases be managed on table. A summary of outcomes can be found in Table 2.

**Table 2:** Summary of outcomes of thoracic aortic stent grafting

	DTAA (rupture)	DTAA (rupture and elective)	TBAD	BTAI
Technical success	91.9–100%	91.9–100%	91.9–100%	91.9–100%
In-hospital mortality			2.6–9.8%	9% (27% for surgery)
30-day mortality	5.57% (16.5% for surgery)	5.57% (16.5% for surgery)	5.57% (16.5% for surgery)	5.57% (16.5% for surgery)
	19% (33% for surgery)	4.4% (3.2% for surgery)		
1-year mortality	22% (24% for surgery)	22% (24% for surgery)	4.7% (6% for medical management)	
5-year mortality	44.3% (37.4% for surgery)	44.3% (37.4% for surgery)		
5-year survival				88.9% (65.1% for surgery)
Major Complications			3.4–11.1%	
Major neurological injury	0.6–5.4% (14% for surgery)	0.6–5.4% (14% for surgery)	0.6–5.4% (14% for surgery)	0.6–5.4% (14% for surgery)
Re-intervention	2.6–7% (8.4% for surgery)	2.6–7% (8.4% for surgery)	2.6–7% (8.4% for surgery)	2.6–7% (8.4% for surgery)
		6.7%	8.1–11.5% (27.7% for medical management)	1.8%
Length of hospital stay			11.1 days (6.4 days for medical management)	

*DTAA* Descending thoracic aortic aneurysm*; TBAD* Type B aortic dissection*; BTAI* Blunt traumatic aortic injury

### Clinical Outcomes

TEVAR for all types of thoracic aortic disease has been shown to have a low 30-day mortality (5.57%), with low rates of neurological injury (5.4%) and major re-intervention (7%). This compares favourably with open surgery (16.5% mortality, 14% neurological injury, 8.4% re-intervention), with a reduced length of hospital and critical care stay also reported for TEVAR (minus 1 day and minus 1.26 days, respectively) [52].

#### Descending Thoracic Aortic Aneurysms

In ruptured DTAA, 30-day mortality for TEVAR versus open surgery has been reported as 19% vs. 33%, respectively, with TEVAR also associated with lower rates of myocardial infarction (MI), stroke, and paraplegia [53]. In TEVAR for mycotic DTAA, mortality is reported as 8% at 30 days and 22% at 1 year [4] but does carry the specific risks of persistent sepsis, re-infection, and the necessity of prolonged antibiotic therapy.

An analysis of TEVAR vs open surgery in 14,580 DTAA patients (emergency and elective) showed slightly higher operative mortality for TEVAR (4.4% vs. 3.2%) and higher vascular complications (5.3% vs. 1.2%), but no difference in 1- and 5-year mortality (22.2% vs. 24% and 44.3% vs. 37.4%, respectively) [54]. Additionally, TEVAR was associated with much shorter intensive care and hospital stays, and fewer non-vascular complications (paraplegia, renal failure, and cardiac complications).

#### Type B Aortic Dissection

Studies looking specifically at TEVAR for TBAD have reported in-hospital mortality rates of 2.6–9.8%, with major complication rates of 3.4–11.1%, including neurological complication rates of 0.6–3.1% [5, 55, 56]. Another study reported 1-year mortality for TEVAR versus medical management in acute uTBAD patients and found no significant difference (4.7 and 6%, respectively). Significant differences were the longer length of stay in hospital post-TEVAR (11.1 days vs. 6.4), but lower rates of future emergency operations (8.1% vs. 27.7%) [51].

#### Trauma

In-hospital mortality post-TEVAR for traumatic aortic injury is reported at 9% (significantly lower than open surgery: 27%), with a 5-year survival of 88.9% (vs. 65.1% for open surgery) [57].This study, which looked retrospectively at 287 trauma patients, also reported post-TEVAR freedom from intervention as 97.4% (vs. 100% for open surgery).

### Re-intervention

Re-interventions are not uncommon and are indicated for managing changes related to remodelling, endoleak, and subsequent vascular injury. Risk factors for post-TEVAR re-intervention include proximal aortic involvement (zone 0) and larger graft diameter, along with other indicators of complexity: greater contrast volume, longer procedure time, arm/neck access, greater number of aortic devices, and end of procedure endoleak [58]. A review of 7006 TBAD patients from the Vascular Quality Initiative data in the USA has shown an 11.5% re-intervention rate for TBAD, 6.7% for TAA, and 1.8% for trauma [58]. The most common indications were type 1 and 2 endoleaks, malperfusion, false lumen perfusion, extension of dissection, and, in the longer term, aortic dilatation. Patients undergoing a re-intervention (for TBAD and TAA) had no reduction in survival compared with those who did not receive an intervention; however, those who required in-hospital re-intervention did tend towards decreased survival. The most common re-interventions included further stent grafting or bare stenting, and endovascular or surgical treatment of a branch.

### Complications

#### Stent Graft-Induced New Entry

SINE is the formation of a new intimal tear arising as a complication of stent deployment, which can be retrograde or antegrade. Retrograde dissections occur at the proximal end of the stent graft and extend back into the arch or ascending aorta. It occurs more often in TBAD than TAA (4.7–5.1% vs. 0.66%) and carries significant risk (37.1% mortality) [58]. Proximal placement of the stent graft is associated with an increased rate of retrograde dissection: 2.57% in zone 2, decreasing to 0.67% in zone 3–4 [58, 59]. Antegrade aortic dissection in TBAD has an incidence of 10.1%, with excessive oversizing and chronic dissection being significant risk factors.

#### Stroke

Post-TEVAR stroke rates have been reported at 4.1–4.4% [60, 61]. It is not an unexpected complication of TEVAR given the manipulation of devices across the aortic arch (which should be minimised) and also the potential for occlusion of the LSA. When LSA coverage is performed, the stroke rate increases to 8–11.8%, reducing to 2.8–5.3% if LSA revascularisation is performed [60, 61]. Other factors known to significantly increase the risk of stroke include debranching procedures, proximal stent landing zones (zones 0–2: covering brachiocephalic trunk, left common carotid, or LSA), and patients with high grade atheroma [50]. There is a higher incidence of non-clinically significant cerebral infarction, with a small-scale study suggesting that this can be up to 34.4% but with only an associated 1.6% clinical stroke rate [50].

#### Spinal Cord Ischaemia

TEVAR graft coverage of the LSA and intercostal arteries limits blood supply to the spinal cord and can lead to transient or permanent symptoms of SCI. In TEVAR for TAA, a SCI incidence of 11% is reported (with permanent symptoms in 6%). In addition to basic measures (including pre-procedure correction of anaemia and maintenance of good oxygen saturation), reduced SCI rates are observed with shorter graft lengths and staged procedures [62].

#### Infection

Stent graft infection is an uncommon (0.2–0.7%) but significant complication of TEVAR [63], and survival rates are poor (27% late survival) [64]. Diagnosis is made by clinical presentation (sepsis, rupture, aorto-enteric fistulae), imaging features (CT, PET, leucocyte studies), and microbiology from blood cultures. Treatment relies on surgical explantation of the graft, or conservative management with antibiotics if the patient is not a surgical candidate [64].

#### Post-Implantation Syndrome

Post-implantation syndrome is a non-infectious inflammatory syndrome, classically consisting of fever, raised inflammatory markers, and coagulation disorder in the absence of positive blood cultures. Incidence ranges from 14 to 28%, but with no significant effect on survival [65]. Infection is an important differential in these situations and should be excluded.

## Conclusion

TEVAR is an established standard of care for treatment for thoracic aneurysm, AAS, and traumatic injury. Careful patient selection, imaging, and treatment planning, along with a comprehensive understanding of treatment risks and complications along with their mitigating measures, is critical to ensuring good outcomes.

## Abbreviations

AAS: Acute aortic syndrome
BTAI: Blunt traumatic aortic injury
CSF: Cerebrospinal fluid
DTA: Descending thoracic aorta
DTAA: Descending thoracic aortic aneurysm
DTAD: Descending thoracic aortic disease
IMH: Intramural haematoma
LSA: Left subclavian artery
MAP: Mean arterial pressure
MI: Myocardial infarction
PAU: Penetrating aortic ulcer
SCI: Spinal cord ischaemia
SINE: Stent graft-induced new entry
TAA: Thoracic aortic aneurysm
TBAD: Type B aortic dissection
cTBAD: Complicated TBAD
uTBAD: Uncomplicated TBAD
TEVAR: Thoracic endovascular aortic repair
